# The absolute risk of gout by clusters of gout-associated comorbidities and lifestyle factors—30 years follow-up of the Malmö Preventive Project

**DOI:** 10.1186/s13075-020-02339-0

**Published:** 2020-10-16

**Authors:** Tahzeeb Fatima, Peter M. Nilsson, Carl Turesson, Mats Dehlin, Nicola Dalbeth, Lennart T. H. Jacobsson, Meliha C. Kapetanovic

**Affiliations:** 1 Department of Rheumatology and Inflammation Research, Sahlgrenska Academy, University of Gothenburg, Gothenburg, Sweden; 2 Lund Arthritis Research Group, Lund University, Lund, Sweden; 3 Department of Clinical Sciences, Lund University, Malmö, Sweden; 4 Rheumatology, Department of Clinical Sciences, Lund University, Malmö, Sweden; 5 Department of Rheumatology, Skåne University Hospital, Malmö, Sweden; 6 Department of Medicine, University of Auckland, Auckland, New Zealand; 7 Department of Clinical Sciences Lund, Section of Rheumatology, Lund University and Skåne University Hospital, Lund, Sweden

**Keywords:** Gout, Urate, Epidemiology, Comorbidities, Clusters, Risk

## Abstract

**Background:**

Gout is predicted by a number of comorbidities and lifestyle factors. We aimed to identify discrete phenotype clusters of these factors in a Swedish population-based health survey. In these clusters, we calculated and compared the incidence and relative risk of gout.

**Methods:**

Cluster analyses were performed to group variables with close proximity and to obtain homogenous clusters of individuals (*n* = 22,057) in the Malmö Preventive Project (MPP) cohort. Variables clustered included obesity, kidney dysfunction, diabetes mellitus (DM), hypertension, cardiovascular disease (CVD), dyslipidemia, pulmonary dysfunction (PD), smoking, and the use of diuretics. Incidence rates and hazard ratios (HRs) for gout, adjusted for age and sex, were computed for each cluster.

**Results:**

Five clusters (C1–C5) were identified. Cluster C1 (*n* = 16,063) was characterized by few comorbidities. All participants in C2 (*n* = 750) had kidney dysfunction (100%), and none had CVD. In C3 (*n* = 528), 100% had CVD and most participants were smokers (74%). C4 (*n* = 3673) had the greatest fractions of obesity (34%) and dyslipidemia (74%). In C5 (*n* = 1043), proportions with DM (51%), hypertension (54%), and diuretics (52%) were highest. C1 was by far the most common in the population (73%), followed by C4 (17%). These two pathways included 86% of incident gout cases. The four smaller clusters (C2–C5) had higher incidence rates and a 2- to 3-fold increased risk for incident gout.

**Conclusions:**

Five distinct clusters based on gout-related comorbidities and lifestyle factors were identified. Most incident gout cases occurred in the cluster of few comorbidities, and the four comorbidity pathways had overall a modest influence on the incidence of gout.

## Background

The development of gout is influenced by multiple genetic and environmental risk factors and interactions among these [1, 2]. The symptoms of gout arise from the body’s innate immune response to monosodium urate crystals that accumulate in the joints, tendons, and other tissues as a result of hyperuricemia (HU), which is defined as serum urate (SU) level > 408 μmol/L [3]. The global prevalence of gout is gradually increasing and ranges from 1 to 4% in developed countries [4]. In Sweden, regional studies have shown a prevalence of approximately 1.7% and that gout is 2 to 3 times more common in men than in women [5–7].

Gout is associated with several comorbidities and lifestyle factors. The increase in gout prevalence over the last decades is partly attributed to aging of the general population and the rise of comorbid conditions such as obesity and associated insulin resistance, as well as changing lifestyle and dietary practices [3]. The prevalence of comorbid conditions such as hypertension, obesity, CVD, diabetes, dyslipidemia, and chronic kidney disease (CKD) is significantly higher in people with gout than in the general population [8]. A recent study from the USA indicated an increase in gout incidence over 20 years and a multifold increase in many associated comorbidities in patients with gout during this time period. The relative increase was 3-fold for renal disease and dyslipidemia, 4-fold for diabetes, 30% for hypertension, and 50% for obesity [9]. The close correlation between several of these comorbidities makes it a challenge to examine their individual importance as risk markers for HU and gout using the traditional epidemiological analyses. Furthermore, HU and gout may also be potential risk factors for hypertension, CVD, CKD, and the metabolic syndrome [8, 10]. In studies using Mendelian randomization (MR) analyses, there is some support for obesity as a causal risk factor for HU and gout [11], whereas such studies have so far failed to support causality between HU and/or gout and other risk factors including CVD, CKD, and diabetes [12–14].

Gout has also been associated with several lifestyle factors including alcohol consumption and smoking. Whereas increased alcohol consumption has been suggested to elevate SU concentrations and to act as a trigger of acute gout attacks [15, 16], the relative importance of smoking is slightly controversial with different studies providing conflicting results [17, 18]. Epidemiological studies, examining the effect of different diuretics on the risk of gout, collectively support that such medication could increase the incidence of gout [19, 20].

Since these factors (comorbidities and lifestyle) often are closely associated with each other, they may represent a few pathophysiological pathways rather than being individually important predictors. Identifying clusters of such factors may thus lead to a better understanding of the potential pathways involved in the risk of gout. Two studies have previously reported four to five phenotype clusters in prevalent cohorts of gout patients of European ancestry [21, 22]. However, the identification of such clusters (generalizability of the pattern analysis) has neither been assessed in the general population, nor to what extent they predict future gout.

The purposes of our observational, prospective study were to (1) identify clusters of gout-related baseline comorbidities and lifestyle factors among the participants of a population-based health survey and (2), for each of these identified clusters, determine absolute and relative risks for developing gout during 30 years of follow-up.

## Methods

### Study cohort

The study used baseline and follow-up data from the Malmö Preventive Project (MPP) cohort (Additional file 1: Figure S1). The MPP was a population-based screening program for cardiovascular risk factors, alcohol abuse, and breast cancer that started in 1974 at the Department of Preventive Medicine, University Hospital, Malmö, Sweden. Individuals born in Malmö certain years and residents of the city were invited for a clinical examination, questionnaire, and blood sampling. A total of 33,346 (22,444 men and 10,902 women) individuals, aged 26 years and over, participated and were screened at the baseline between 1974 and 1992. During the first half of the period (1974–1982), mostly men and, during the second half (1982–1992), mostly women were invited to participate. The vast majority of the participants were Caucasians of Scandinavian origin. More details on the cohort, individual questionnaire, and data recruitment for the MPP cohort are provided elsewhere [23].

The Regional Ethics Committee at Lund University (Dnr 85/2004) provided ethical approval for this follow-up study.

### Endpoint retrieval of gout

Date of the gout diagnosis was defined as the first visit with a registered diagnosis of gout [using International Classification of Diseases (ICD) versions 8, 9, and 10 (Additional file 1: Table S1)] to physicians in primary health care (from 1998), specialized inpatient care (from 1974), or outpatient care (1998 onward). The date for gout diagnosis was obtained through linkage of MPP cohort with regional (The Skåne Healthcare Register) and national health care (National Patient Register) registers. For the longitudinal analyses, follow-up started at the time point of baseline screening, which could vary between 1974 and 1992. Participants were followed until the date of first gout diagnosis, death, migration from the area, or December 31, 2014, whichever occurred first. The individuals were thus followed for a period of up to 30 years (mean 28.2 ± 8.4 years).

For this study, the baseline data from the health examination performed anytime between 1974 and 1992 for various gout-related comorbidities and lifestyle factors were used from the MPP cohort. Obesity was defined as body mass index (BMI) > 30 kg/m^2^. Kidney dysfunction was defined as a glomerular filtration rate (eGFR) of less than 60 mL/min/1.73m^2^. Diabetes mellitus was defined as single fasting blood glucose ≥ 6.7 mmol/L (according to contemporary reference levels in the 1970s) or a history of DM. Individuals with repeated systolic blood pressures ≥ 160 mmHg and/or diastolic blood pressures > 100 mmHg, or those who reported being on antihypertensive therapy, were considered to have hypertension [23]. Prevalent CVD was defined as a history of angina pectoris, myocardial infarction, or concomitant medication for heart disease. Dyslipidemia was defined by the presence of either hypercholesterolemia (serum cholesterol > 6.1 mmol/L) or hypertriglyceridemia (serum fasting triglyceride > 2.6 mmol/L). Pulmonary dysfunction (PD) was defined by measured spirometry values of the forced vital capacity (FVC) and the forced expiratory volume within 1 s (FEV_1_) as FEV_1_/FVC < 70% of predicted values [24]. Exposure to smoking status was considered present if subjects were current smokers or had a recent history of at least 10 years of smoking. The use of diuretics was based on the information provided by the participants in the study questionnaire. The information for SU and liver enzyme concentrations was also measured at baseline at the Department of Clinical Chemistry in Malmö [23]. The term “abnormal liver enzymes” was defined by the presence of serum alanine aminotransferase > 1.1 μkat/L (for men) and > 0.75 μkat/L (for women) and/or serum gamma-glutamyl transferase > 1.3 μkat/L (for men) and > 0.75 μkat/L (for women). Alcohol consumption behavior (risk behavior) was assessed using the Malmö-modified brief Michigan Alcohol Screening Test (Mm-MAST) comprising nine questions regarding alcohol habits [25]. According to previous validation studies, a score of ≥ 2 was classified as a risk drinking behavior [26]. All individuals who reported to have prevalent gout (*n* = 11) before or at the time of inclusion in the MPP were excluded from further analyses.

### Statistical analysis

The study mainly involved cluster and proportional hazard analyses in the MPP cohort using the baseline and follow-up data, respectively. Summary data were expressed as mean ± SD for age and BMI, and as frequency and percentages for all categorical variables.

Based on the availability of complete data at baseline for a sufficient number of individuals and based on factors previously being identified as associated with gout in cross-sectional cluster analyses [21, 22], a set of nine variables was selected for the cluster analysis that included gout-related comorbidities and lifestyle factors. Due to the substantial proportions with missing data (questions not asked and tests not performed some years) and reduction in the total variance explained, respectively, for alcohol consumption behavior and information for abnormal liver enzymes, these variables were not included in the main cluster analysis. However, their demographics were calculated for each cluster post-analysis. The individuals included in the cluster analysis were compared with the rest of the population (with incomplete data, not included in the analyses) for age, sex, BMI, and SU levels to evaluate representativeness (Additional file 1: Table S2).

The selected variables were first arranged into homogeneous clusters to obtain a meaningful inter-variable relationship structure using the ClustOfVar package in R v3.5.2 [27]. This package can efficiently cluster categorical variables using principal component analysis based on the correlation ratio of variables with the first component (the cluster center) to create a hierarchy. In addition, a factorial analysis with oblique rotation (direct oblimin; assumes correlated factors) was conducted to establish a factor structure for the selected variables. Only the factors with eigenvalue > 1 were extracted. The highest loading value of a variable (comorbidity or lifestyle) was the inclusion criterion for that particular variable in any factor. The Kaiser-Meyer-Olkin (KMO > 0.5) test was performed to assess the suitability of the data for the factor analysis. Bartlett’s test for sphericity was used to compare the variable correlation matrix to the identity matrix using a standard *p* < 0.05 as a cutoff. The factorial analysis was carried out in SPSS Statistics v25 (IBM Corp., Armonk, NY, USA).

The individual observations were also clustered using the hierarchical agglomerative clustering in R v3.5.2 [28]. The analysis was not forced to provide a fixed number of clusters. Ward’s minimum variance criterion was applied to group the observations based on similarities in comorbidity and lifestyle variables. This method minimizes the total within-cluster variance. The clustering algorithm started with each cluster as a singleton, i.e., cluster containing one single point/observation. The algorithm progressed via finding another cluster to pair with while keeping a minimum increase in total within-cluster variance sum of squares at each step. The process of pairing iterated until all observation points were members of one single cluster.

For all identified clusters, the cumulative incidence as well as the incidence rates of gout was computed with 95% confidence intervals (95% CIs). Using the largest cluster as reference, the Cox proportional hazard model was applied, and both unadjusted and age- and sex-adjusted HRs with 95% CIs were calculated. Student *t* test was used to calculate sex-stratified differences of mean SU concentration for gout case-control groups in each cluster. The cumulative incidence for eight predefined gout-related comorbidities (see Additional file 1: Table S1 for ICD-8, ICD-9, and ICD-10 codes to define these comorbidities) before gout diagnosis was also calculated. The end of follow-up was defined as the date of the first diagnosis for the comorbidity of interest or gout, death, migration from the area, or study end date, i.e., December 31, 2014 (whichever occurred first).

## Results

A subset of 22,057 MPP individuals (screening period 1975–1992) had sufficiently complete data and were eligible for the further classification process. Individuals in this subset were mostly males (66%), with the mean for age and BMI of 46.8 ± 5.5 years and 24.6 ± 3.5 kg/m^2^, respectively. The comorbidities were overall not very frequent, as expected, considering the mean age at baseline and that data was obtained from a general population sample (Table 1). Those included in the analyses had similar values for age, BMI, and urate, but tended to be older (46.8 vs. 43.4 years) and less often men (66% vs. 70%) compared to the rest of the MPP cohort (*n* = 11,278) (Additional file 1: Table S2).

**Table 1:** Characteristics of the individuals in the MPP cohort overall and for the five identified clusters of gout-related comorbidities

Characteristics	All	C1: few comorbidities	C2: CKD/kidney dysfunction	C3: CVD and lifestyle	C4: obesity and dyslipidemia	C5: DM and hypertension
**Total number**	22,057	16,063	750	528	3673	1043
**Demographics**						
**Male,** ***n*** **(%)**	14,561 (66.01)	10,425 (64.90)	420 (56.00)	358 (67.80)	2769 (75.38)	589 (56.47)
**Age (years)**	46.81 ± 5.54	46.21 ± 5.88	51.75 ± 5.67	48.66 ± 2.51	47.47 ± 4.34	48.54 ± 5.31
**BMI**	24.57 ± 3.53	23.47 ± 3.22	24.50 ± 2.87	23.00 ± 4.18	25.91 ± 4.01	25.07 ± 4.23
**Comorbidities**						
**History of kidney stones**	1151 (5.26)	780 (4.90)	52 (7.03)	36 (6.89)	205 (5.61)	78 (7.66)
**Obesity**	1599 (7.24)	0 (0)	66 (8.80)	60 (11.36)	1250 (34.03)	223 (21.38)
**Kidney dysfunction**	876 (3.97)	0 (0)	750 (100)	35 (6.62)	0 (0)	91 (8.72)
**Diabetes mellitus**	537 (2.43)	0 (0)	0 (0)	0 (0)	0 (0)	537 (51.48)
**Hypertension**	4081 (18.50)	2255 (14.03)	204 (27.20)	184 (34.84)	879 (23.93)	559 (53.59)
**CVD**	631 (2.86)	0 (0)	0 (0)	528 (100)	0 (0)	103 (9.87)
**Hypercholesterolemia**	2185 (9.91)	0 (0)	91 (12.13)	74 (14.01)	1888 (51.40)	132 (12.65)
**Hypertriglyceridemia**	1459 (6.61)	0 (0)	49 (6.53)	56 (10.61)	1166 (31.74)	188 (18.02)
**Dyslipidemia**	3217 (14.58)	0 (0)	124 (16.53)	114 (21.59)	2712 (73.84)	267 (25.59)
**Abnormal liver enzymes**	1718 (7.78)	934 (5.81)	54 (7.20)	51 (9.65)	507 (13.80)	172 (16.49)
**Pulmonary dysfunction**	3451 (15.64)	2425 (15.09)	128 (17.06)	119 (22.53)	595 (16.19)	184 (17.64)
**Lifestyle/medication**						
**Smoking**	13,083 (59.31)	9295 (57.86)	406 (54.13)	392 (74.24)	2380 (64.79)	610 (58.48)
**Alcohol risk behavior**	4538 (29.79)	3136 (28.02)	116 (25.27)	138 (40.82)	943 (36.27)	205 (31.88)
**Use of diuretics**	541 (2.45)	0 (0)	0 (0)	0 (0)	0 (0)	541 (51.87)

The characteristics are presented as mean ± standard deviation for continuous variables and number (percentages) for categorical variables. C1 to C5 represent cluster numbers 1 to 5. *n* total number, *CKD* chronic kidney disease, *CVD* cardiovascular disease, *DM* diabetes mellitus

### Clustering of comorbidities and lifestyle variables

**Fig. 1:**
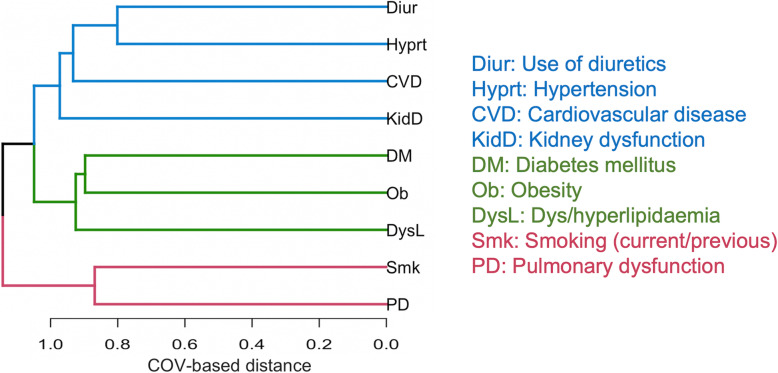
Dendrogram illustrating the results of variable clustering using the ClustOfVar package. Variables with the closest proximity were grouped, where proximity was based on similarity/difference in the pattern of individual’s response to any variable. Variables are represented by horizontal lines, and the length of horizontal lines represents the degree of similarity between variables. The “COV-based distance” on the *x*-axis indicates values for a distance metric between variables and clusters calculated by ClustOfVar (COV). Each color (blue, green, and red) represents a different cluster

The variables selected for clustering included seven gout-related comorbidities: obesity, kidney dysfunction, DM, hypertension, CVD, dyslipidemia, PD, one lifestyle factor (smoking), and the use of diuretics. The variables were not completely uncorrelated; however, the correlation was very low, never exceeding 0.20 (Additional file 1: Figure S2). Initial clustering indicated three clusters with discrete patterns: CV-1 that grouped CVD, kidney dysfunction, hypertension, and use of diuretics; CV-2 clustered DM, obesity, and dyslipidemia, whereas PD and smoking formed CV-3 (Fig. 1). Factorial analysis extracted three factors (F1, F2, F3) accounting for 41.3% of the variance and confirming the results of cluster analysis. F1 combined cardiovascular risk factors and renal disease (CVD, kidney dysfunction, hypertension, and use of diuretics), F2 combined variables associated with pulmonary etiologies (PD and smoking), and F3 consisted of variables associated with the insulin resistance (DM, obesity, and dyslipidemia) (Additional file 1: Table S3). The correlation matrix indicated a weak correlation (*p* < 0.009) and a good separation between the factors. The KMO statistic was 0.58, with Bartlett’s test being significant (*p* < 0.0001), indicating that factor analysis was appropriate.

### Clustering of data observations

**Fig. 2:**
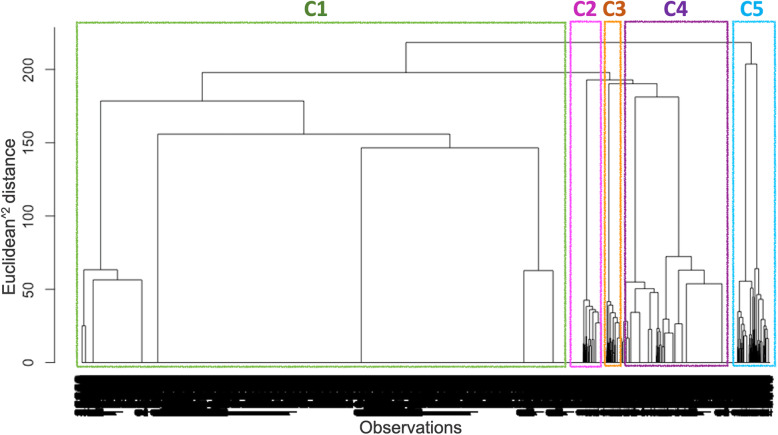
Dendrogram illustrating the results of cluster analysis in data observations (*n* = 22,057). The vertical axis on the graph represents the distance between clusters, while the horizontal axis represents the observations and clusters. Each vertical bar represents a subject and a cluster, and joining of two clusters is represented by the fusion of two vertical bars. The vertical position of the fusion, shown by short horizontal lines, gives the distance between the two clusters. C1 to C5 represent cluster numbers 1 to 5

The same set of selected variables (comorbidities and lifestyle/medication factors) were used to cluster individual observations. The clustering analyses resulted in five different clusters: C1 to C5 (Fig. 2). The baseline characteristics of these clusters are given in Table 1.

The first cluster (C1; *n* = 16,063) was the largest, which entailed 73% of the population with a mean age of 46 years and low frequencies for comorbidities. Almost all individuals in this cluster were healthy, i.e., none had obesity, kidney dysfunction, DM, CVD, or dyslipidemia. Only small proportions had hypertension (14%) or PD (15%). This cluster was labeled as “few comorbidities.”

Individuals in C2 (*n* = 750, 3.4%) were the oldest with a mean age of 51 years. All individuals in this cluster had kidney dysfunction (100%), and none had DM, CVD, or used diuretics. This cluster was labeled as “CKD/kidney dysfunction.”

In C3 (*n* = 528; 2.4%, mean age 48 years), all individuals had CVD (100%), and the group had the highest proportion of PD (22%), smoking (74%), and alcohol risk behavior (40%). None in this cluster had DM or used diuretics. The cluster was labeled as “CVD and lifestyle.”

Cluster C4 (*n* = 3673; mean age 47 years) represented the second-largest proportion (17%) of the clustered observations. Individuals in C4 were mostly males (75%) and had the highest mean BMI (25.9 kg/m^2^) and the largest proportions with obesity (34%) and dyslipidemia (74%) [hypercholesterolemia: 51% and hypertriglyceridemia: 32%]. This cluster was labeled “obesity and dyslipidemia.”

Cluster C5 (*n* = 1043; mean age 48 years) had by far the highest occurrence of DM (51%), hypertension (54%), and use of diuretics (52%), and the highest percentage of those with abnormal liver enzyme levels (16%). C5 was labeled “diabetes and hypertension.”

### Risk of developing gout

For the subset of 22,057 MPP participants (mean follow-up of 28.1 (95% CI 27.9 to 28.2) years) included in the cluster analysis, a total of 910 (4.1%) developed incident gout after a mean follow-up of 27.5 years (range between clusters, 23.4 to 28.2; Table 2). The absolute risk of incident gout, and unadjusted and age- and sex-adjusted HRs are given in Table 2. The majority of incident gout cases during follow-up came from the largest cluster, C1 (“few comorbidities,” 60%) followed by C4 (“obesity and dyslipidemia,” 26%). The incidence of gout was highest for C2 (276.8 per 100,000 person-years at risk), and the highest relative risk for gout development was seen for C2 [HR (95% CI) 2.31 (1.73 to 3.07)] and C3 [HR (95% CI) 2.41 (1.63 to 3.58)]. However, all four clusters (C2–C5) identified by different comorbidity patterns resulted in a 2- to 3-fold increased risk for incident gout compared to C1 (Table 2).

**Table 2:** Incidence and hazard ratios (HRs) for gout during follow-up and mean SU of diagnosed patients with gout in the five observation-clusters of the MPP cohort

Cluster number	C1	C2	C3	C4	C5
**Whole cohort (*n*** **= 22,057)**					
**Prevalence**	16,063 (72.82)	750 (3.40)	528 (2.39)	3673 (16.65)	1043 (4.72)
SU mean*	288.94 ± 67.35	322.98 ± 78.88	316.17 ± 79.64	328.49 ± 73.77	318.55 ± 92.40
**Incident cases with gout (*n*** **= 910)**					
**Incident gout**	551 (60.54)	53 (5.82)	26 (2.85)	235 (25.82)	45 (4.94)
**SU mean**^**°**^	348.05 ± 72.51	384.35 ± 85.67	357.19 ± 96.18	384.74 ± 77.59	389.17 ± 94.96
**Age at gout diagnosis**	74.79 ± 6.75	76.92 ± 8.11	74.29 ± 9.67	73.82 ± 7.21	72.73 ± 8.49
**Follow-up time**	28.22 ± 5.52	25.53 ± 6.15	25.19 ± 7.52	27.42 ± 5.99	23.49 ± 8.36
**Incidence and HR for gout**					
**Incidence (95%CI)**^**^**^	121.5 (111.8 to 132.1)	276.8 (194.4 to 394.1)	195.5 (133.1 to 287.0)	233.3 (205.3 to 265.1)	183.6 (137.2 to 245.9)
**HR-unadjusted (95%CI)**	1	2.74 (2.06–3.63)	2.72 (1.83–4.03)	2.18 (1.87–2.54)	2.10 (1.55–2.85)
**HR-adjusted (95% CI)**^**γ**^	1	2.31 (1.73–3.07)	2.41 (1.63–3.58)	2.02 (1.73–2.35)	1.98 (1.46–2.68)

The values are presented as mean ± standard deviation for continuous variables and number (percentages) for categorical variables. Age and the follow-up time are calculated in years. C1 to C5 represent cluster numbers 1 to 5. *SU* serum urate, *HR* hazard ratio, *95% CI* 95% confidence interval

*Baseline serum urate (μmol/L) for clusters in all individuals

^°^Baseline serum urate (μmol/L) for incidence gout group

^^^Incidence per 100,000 person-years at risk

^γ^Adjusted for age and sex

Sex-stratified SU level comparisons between those developing gout and those not during follow-up showed the highest differences for C5 men [Δ (95% CI) 80.3 μmol/L (41.6 to 119.0)] and C2 women [Δ (95% CI) = 73.7 μmol/L (27.6 to 119.7)], while the differences were lowest for C3, in both men [Δ (95% CI) 47.7 μmol/L (11.0 to 84.4)] and women [Δ (95% CI) 30.1 μmol/L (− 13.2 to 73.4)] (Additional file 1: Table S4).

### Incidence of gout-related comorbidities in the five clusters

Comorbidities that are considered to be well-documented risk factors/markers for HU and gout, such as obesity, CKD, and diagnosed alcoholism, had much lower cumulative incidence (2.4%, 19.4%, and 0.4%, respectively) in the “few comorbidities” cluster (C1) compared to other clusters, even when combining those identified at baseline and with a diagnosed disease during follow-up (Table 3). Also, for other comorbidities considered to be associated with HU or gout, C1 had numerically lower (diabetes, dyslipidemia, CVD, hypertension) cumulative incidence compared to other clusters (Table 3), although considerable proportions were diagnosed with hypertension (57%) or CVD (46%) before gout diagnosis. During the follow-up, the cumulative incidence of several comorbidities remained comparatively higher (CKD, 31.1%; dyslipidemia, 17.8%; and COPD, 11.1%) in C5 than in other clusters.

**Table 3:** Within clusters’ cumulative incidence for various gout-related comorbidities for gout incident cases during follow-up (*n* = 910)

Cluster number	C1	C2	C3	C4	C5
**Gout incident cases (*n*)**	551	53	26	235	45
**Diabetes (DM)**					
**Baseline,** ***n*** **(%)**	0 (0)	0 (0)	0 (0)	0 (0)	19 (42.2)
**Incident cases,** ***n*** **(%)**	81 (14.7)	6 (11.3)	2 (7.7)	62 (26.4)	8 (17.8)
**Total cases,** ***n*** **(%)**	81 (14.7)	6 (11.3)	2 (7.7)	62 (26.4)	27 (60.0)
Follow-up time, mean ± SD*	28.45 ± 8.62	25.55 ± 8.81	21.31 ± 10.16	25.63 ± 9.14	17.73 ± 11.24
**Obesity**					
**Baseline,** ***n*** **(%)**	0 (0)	7 (13.2)	3 (11.5)	93 (39.6)	17 (37.8)
**Incident cases,** ***n*** **(%)**	14 (2.5)	0 (0)	2 (7.7)	7 (3.0)	0 (0)
**Total cases,** ***n*** **(%)**	14 (2.5)	7 (13.2)	5 (19.2)	100 (42.5)	17 (37.8)
Follow-up time, mean ± SD*	29.22 ± 8.36	26.48 ± 8.64	22.22 ± 10.29	27.15 ± 9.01	23.46 ± 9.98
**Dyslipidemia**					
**Baseline,** ***n*** **(%)**	0 (0)	11 (20.8)	6 (23.1)	113 (48.1)	15 (33.3)
**Incident cases,** ***n*** **(%)**	54 (9.8)	4 (7.5)	3 (11.5)	16 (6.8)	8 (17.8)
**Total cases,** ***n*** **(%)**	54 (9.8)	15 (28.3)	9 (34.6)	129 (54.8)	23 (51.1)
Follow-up time, mean ± SD*	29.28 ± 8.36	26.59 ± 8.53	22.26 ± 10.31	27.51 ± 8.88	23.86 ± 9.76
**Hypertension**					
**Baseline,** ***n*** **(%)**	112 (20.3)	18 (34.0)	6 (23.1)	79 (33.6)	23 (51.1)
**Incident cases,** ***n*** **(%)**	201 (36.5)	14 (26.4)	10 (38.5)	75 (31.9)	11 (24.4)
**Total cases,** ***n*** **(%)**	313 (56.8)	32 (60.3)	16 (61.5)	154 (65.5)	34 (75.5)
Follow-up time, mean ± SD*	26.77 ± 8.62	23.28 ± 9.28	19.65 ± 9.83	24.19 ± 9.07	19.29 ± 9.64
**Cardiovascular disease (CVD)**					
**Baseline,** ***n*** **(%)**	0 (0)	0 (0)	26 (100)	0 (0)	4 (8.9)
**Incident cases,** ***n*** **(%)**	253 (45.9)	24 (45.3)	NA	119 (50.6)	21 (46.7)
**Total cases,** ***n*** **(%)**	253 (45.9)	24 (45.3)	26 (100)	119 (50.6)	25 (55.5)
Follow-up time, mean ± SD*	27.27 ± 9.11	24.43 ± 9.15	18.01 ± 10.41	24.29 ± 9.71	20.98 ± 10.17
**Kidney dysfunction and chronic kidney disease (CKD)**					
**Baseline,** ***n*** **(%)**^**^**^	0 (0)	53 (100)	3 (11.5)	0 (0)	7 (15.6)
**Incident cases,** ***n*** **(%)**	107 (19.4)	NA	4 (15.4)	49 (20.9)	14 (31.1)
**Total cases,** ***n*** **(%)**	107 (19.4)	53 (100)	7 (26.9)	49 (20.9)	21 (46.6)
Follow-up time, mean ± SD*	28.03 ± 9.06	24.89 ± 9.47	21.34 ± 10.25	26.22 ± 9.31	22.37 ± 9.90
**Pulmonary dysfunction (PD) and chronic obstructive pulmonary disease (COPD)**					
**Baseline,** ***n*** **(%)**^**^**^	86 (15.6)	7 (13.2)	4 (15.4)	38 (16.2)	10 (22.2)
**Incident cases,** ***n*** **(%)**	37 (6.7)	4 (7.5)	1 (3.8)	19 (8.1)	5 (11.1)
**Total cases,** ***n*** **(%)**	123 (22.3)	11 (20.7)	5 (19.2)	57 (24.2)	15 (33.3)
Follow-up time, mean ± SD*	28.45 ± 8.89	25.85 ± 8.95	21.36 ± 10.47	26.69 ± 9.27	22.83 ± 10.11
**Alcohol risk behavior (ALR) and diagnosed alcoholism (ALC)**					
**ALR at baseline,** ***n*** **(%)**	159 (28.9)	11 (20.8)	8 (30.8)	74 (31.5)	9 (20.0)
**Incident cases with ALC,** ***n*** **(%)**	2 (0.4)	1 (1.9)	0 (0)	2 (0.9)	1 (2.2)
**Total cases,** ***n*** **(%)**	NA	NA	NA	NA	NA
Follow-up time, mean ± SD*	28.81 ± 9.02	26.37 ± 8.81	21.69 ± 10.81	27.06 ± 9.43	23.39 ± 10.16

The characteristics are presented as mean ± standard deviation for continuous variables and number (percentages) for categorical variables. *NA* not applicable, *n* total number, *SD* standard deviation

*Follow-up time is calculated in years

^^^Baseline kidney dysfunction is defined by an eGFR < 60 mL/min/1.73m^2^ while baseline pulmonary dysfunction is defined by a FEV_1_/FVC < 70% of predicted values

## Discussion

In a population-based setting, we identified four clusters with increased occurrence of gout-related comorbidities and one with very few such comorbidities. The latter entailed the vast majority of diagnosed gout disease (60%), whereas the relative risks for the four clusters characterized by various combinations of comorbidities were 2- to 3-fold higher. Two studies to date have performed cluster analyses to categorize comorbidities in prevalent gout patients in European populations, using cross-sectional data [21, 22]. Our study is the first to identify gout-related comorbidities’ clusters as predictors of gout longitudinally in a general population, and to compare the absolute and relative risk for incident gout in such clusters.

A cluster with few comorbidities was also defined in European studies performed in prevalent gout patients, although the proportions were lower, 12% [21] and 36% [22], respectively, compared to ours. In the present study, comorbidities were determined on average 27.5 years before gout diagnosis. None in this cluster characterized by “few comorbidities” had kidney dysfunction, DM, obesity, or dyslipidemia at baseline, and only a minority developed such comorbidities before gout diagnosis. Nevertheless, 3.4% of this cluster was diagnosed with gout during follow-up and constituted 60% of the incident gout cases in our study. This supports the concept that other pathways, not defined by comorbidities, are of major importance for the development of clinical gout. These entail both genetic (in particular for HU) and non-genetic (e.g., diet and environment) pathways. Genome-wide association studies have identified a number of genes directly associated with SU levels and their possible contribution to gout [29, 30]. While the genetics of progression from HU to gout are yet poorly understood, studies have shown genetic variants to account for considerable variance in SU levels (27–41%) [29] and gout risk (30%) [31] in Europeans. The risk attributed to the diet may also be important [32, 33], although there are conflicting results regarding its effect size [34]. There could also be a genetic component to gout independent of other comorbidities. This argument is supported by a more recent study that reported similar genetic risk of gout among three subgroups stratified for BMI [35].

The prevalence of kidney dysfunction was 100% in C2, while none in this group had diabetes or CVD. This cluster has similarities to one of those identified by Bevis et al. [22] in prevalent gout, where 97% had CKD, but very low frequencies of CVD and obesity. In our study, this group characterized by reduced kidney function in midlife had the highest relative risk (HR 2.74) of incident gout, which is in line with the general perception that one out of every ten individuals with CKD develops gout at some stage [36]. We also found that the cumulative incidence of other comorbidities remained comparatively low in this cluster pre-gout diagnosis, except for CVD that was diagnosed in 45% among those who subsequently developed gout group. This is in line with the literature that described cardiovascular complications as a common finding among people with CKD [37, 38].

Associations for CVD with HU and gout have been shown repeatedly in observational studies [39, 40]. Our analysis also revealed a cluster of individuals (C3) who all had CVD, whereas the proportions in other clusters varied from minimal to nil. Interestingly, C3 was the cluster with the least difference in SU between those being later diagnosed vs. not with gout. Studies using the urate-associated genetic variants as instruments (MR studies) have not supported a causal relationship between urate levels and cardiovascular outcomes so far [41, 42]. In addition, causal evidence for cardiovascular disease as a risk factor/marker for gout is also lacking. This cluster also had the highest proportions of subjects who were smokers or had alcohol consumption risk behavior. In C3, the cumulative incidence of other comorbidities remained fairly low pre-gout diagnosis, except for hypertension and dyslipidemia. This cluster seems to represent the combination of a few components of the metabolic syndrome and cardiovascular disease together with lifestyle-related risk factors as possible predictors for gout, for which, so far, a causal relationship is less supported compared to clusters 2 and 4.

The second-largest cluster (C4, 17%) in our population had the highest prevalence of obesity (35%) and dyslipidemia (74%) (hyperlipidemia per se), and an absence of kidney dysfunction, diabetes, or CVD. This is in line with the findings of Richette et al. [21], who identified a cluster in prevalent gout patients with a high occurrence of obesity and with none having diabetes, CVD, or renal failure. C4 also contributed with the second-largest fraction (26%) of incident gout cases in our study. Obesity is a well-recognized risk factor for HU and gout, both based on observational studies [43, 44] and MR studies, having adiposity-associated genetic traits as the exposure for urate levels [45, 46] and gout risk [11]. As expected, C4 entailed the highest cumulative incidence for diabetes (26%) and CVD (50%) before gout diagnosis, compared to other clusters. While insulin resistance can be hypothesized to be a common underpinning link between these three conditions, obesity itself can also be a strong risk factor for cardiovascular events and diabetes. A meta-analysis that included data from over 880,000 individuals from multiple ethnic origins indicated that obesity (determined by BMI-associated genetic variants) could causally increase the risk of type 2 diabetes and coronary artery disease [47].

In C5, all subjects had at least one comorbidity and it was the only cluster that included individuals with DM (51%) and had subjects treated with diuretics (52%). Furthermore, it had the highest baseline prevalence and cumulative incidence (among those developing gout) of hypertension as well as high occurrence of many other comorbidities. Hypertension is common in type 2 diabetes patients, and coexistence of these two conditions worsens the clinical outcomes in humans, especially with increased risks for heart and end-stage renal disease [48]. Mendelian randomization studies have so far not demonstrated a causal relationship between HU and type 2 diabetes [14], although positive associations between insulin resistance-related variants and high urate concentrations and gout risk have repeatedly been shown [49, 50]. The previous two studies [21, 22] in prevalent gout patients also identified clusters with the combination of various comorbidities, albeit with lower proportions of CVD and CKD than in the C5 of our study. Considering the high prevalence of diabetes at baseline, it is not unexpected that among subjects diagnosed with gout during follow-up, this cluster had the highest cumulative incidence of hypertension and dyslipidemia. In C5, it may thus be that a combination of interlinked metabolic conditions, including hypertension, insulin resistance, and cardio-renal diseases, as well as PD favored the onset of gout. However, the exclusive occurrence of subjects at baseline treated with diuretics, a well-known predictor of new-onset as well as recurrence of established gout [43, 51], suggests that this may also be a major contributor to incidence gout via secondary HU in this cluster.

The study has several strengths. First, we were able to largely reproduce at a population level the clusters of gout-related comorbidities previously identified only in prevalent gout patients [21, 22]. Second, the study is based on a large population-based cohort which strengthen the generalizability of these clusters. Third, loss to follow-up was minimal due to the almost complete national Swedish register data. Fourth and most importantly, the longitudinal approach with assessment of comorbidity patterns before gout diagnosis, which has not been used in previous cluster analyses of comorbidities in prevalent gout patients [21, 22], gave the unique opportunity to study both the absolute and relative risk for gout in relation to these comorbidity clusters.

There are also possible limitations of the study. First, there may be misclassification of gout diagnosis. However, previous validation studies suggest a high positive predictive value for gout diagnosis in the Swedish health care registers [52, 53]. Second, those not included in the analyses due to missing baseline covariates may have introduced a selection bias. Since those not included in the analyses had similar BMI and SU to those in the analyses, this is unlikely to have caused major effects on the results. Third, longitudinal information on some exposures (i.e., diuretics, BMI, and SU) during the follow-up would have been useful.

## Conclusions

This population-based study in middle-aged subjects demonstrated the occurrence of five clusters of gout-related comorbidities in the general population. Although clusters representing various comorbidity pathways significantly increased the relative risk for incident clinical gout, the vast majority of gout patients came from the cluster reflecting few comorbidities. These findings support that major determinants for gout include factors other than comorbidities, such as genetics or other environmental factors.

## Supplementary information

**Additional file 1.** Supplemental material. Figure S1 to S2 and Tables S1 to S4.

## Data Availability

Please contact author for data request.

## Abbreviations

BMI: Body mass index
C1 to C5: Cluster 1 to 5
CI: Confidence interval
CKD: Chronic kidney disease
COPD: Chronic obstructive pulmonary disease
CV-1 to CV-3: Variable cluster 1 to 3
CVD: Cardiovascular disease
DM: Diabetes mellitus
eGFR: Estimated glomerular filtration rate
F1 to F3: Factor 1 to 3
FEV1: Forced expiratory volume within 1 s
FVC: Forced vital capacity
HR: Hazard ratio
HU: Hyperuricemia
ICD: International Classification of Diseases
KMO: Kaiser-Meyer-Olkin
Mm-MAST: Malmö-modified brief Michigan Alcohol Screening Test
MPP: Malmö Preventive Project
MR: Mendelian randomization
PD: Pulmonary dysfunction
SD: Standard deviation
SU: Serum urate
