# Supplementary material for: The absolute risk of gout by clusters of gout-associated comorbidities and lifestyle factors—30 years follow-up of the Malmö Preventive Project
Source: Arthritis Res Ther. 2020 Oct 16;22:244. doi: 10.1186/s13075-020-02339-0 (PMC7566061; doi:10.1186/s13075-020-02339-0)
Supplement: Supplementary file 1 — Additional file 1. Supplemental material. Figure S1 to S2 and Tables S1 to S4. [file 13075_2020_2339_MOESM1_ESM.docx]

**Additional file 1**

**The absolute risk of gout** **by clusters of gout-associated comorbidities and lifestyle factors – 30 years follow-up of the Malmö Preventive Project**

**Authors:** Tahzeeb Fatima^1,2^, Peter M. Nilsson^3^, Carl Turesson^4^, Mats Dehlin^1^, Nicola Dalbeth^5^, ^[[1]](#footnote-1)^*Lennart T.H. Jacobsson^1^, ^*^Meliha C. Kapetanovic^6^

**Affiliations:** ^1^Department of Rheumatology and Inflammation Research, Sahlgrenska Academy, University of Gothenburg and ^2^Lund Arthritis Research Group, Lund University, Sweden, ^3^Department of Clinical Sciences, Lund University, Malmö, Sweden, ^4^Rheumatology, Department of Clinical Sciences, Malmö, Lund University and Department of Rheumatology, Skåne University Hospital, Malmö, Sweden, ^5^Department of Medicine, University of Auckland, Auckland, New Zealand, ^6^Department of Clinical Sciences Lund, Section of Rheumatology, Lund University and Skåne University Hospital, Lund, Sweden.

**Corresponding author:** Tahzeeb Fatima, PhD, Department of Rheumatology and Inflammation Research, Sahlgrenska Academy, University of Gothenburg and Lund Arthritis Research Group, Lund University, Sweden.

Telephone: +46 313422491

Fax: +46 31823925

Email: [tahzeeb.fatima@gu.se](mailto:tahzeeb.fatima@gu.se)

**Supplemental material**


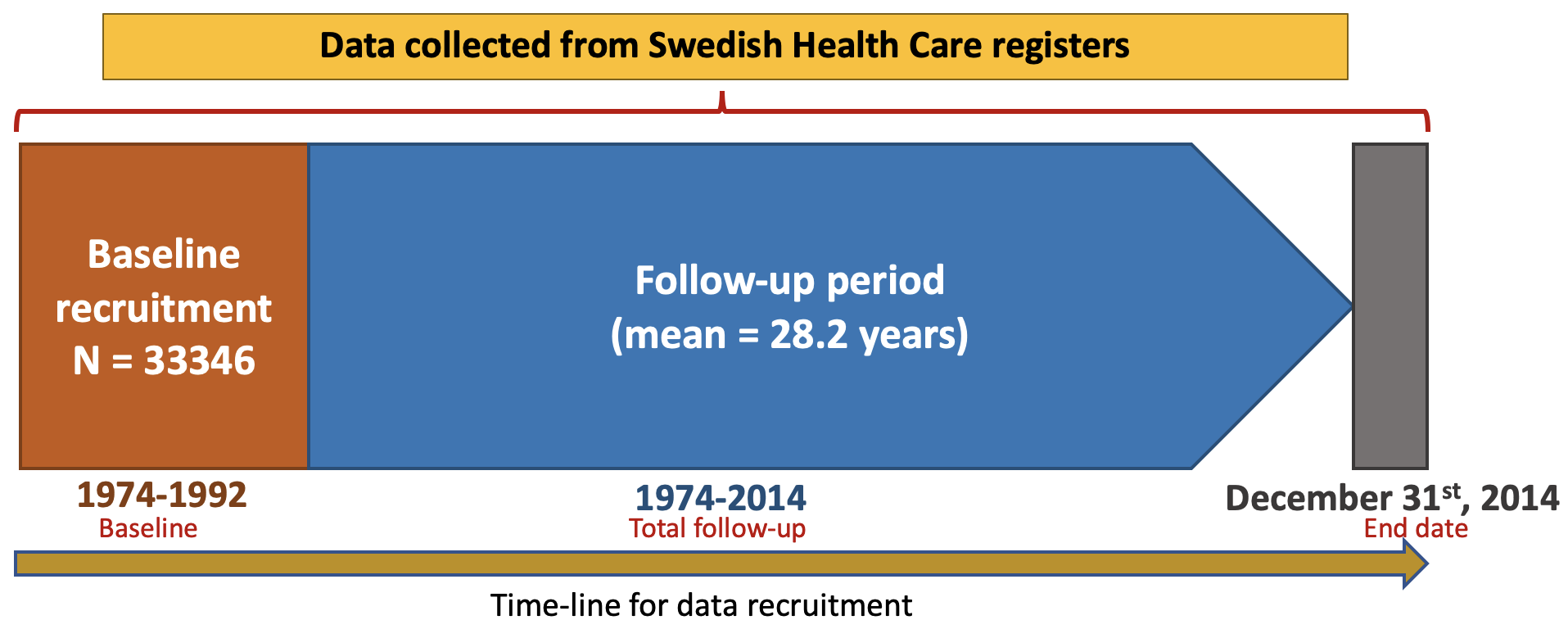


Figure S1 Baseline recruitment and follow-up period for the Malmö Preventive Project (MPP) cohort.


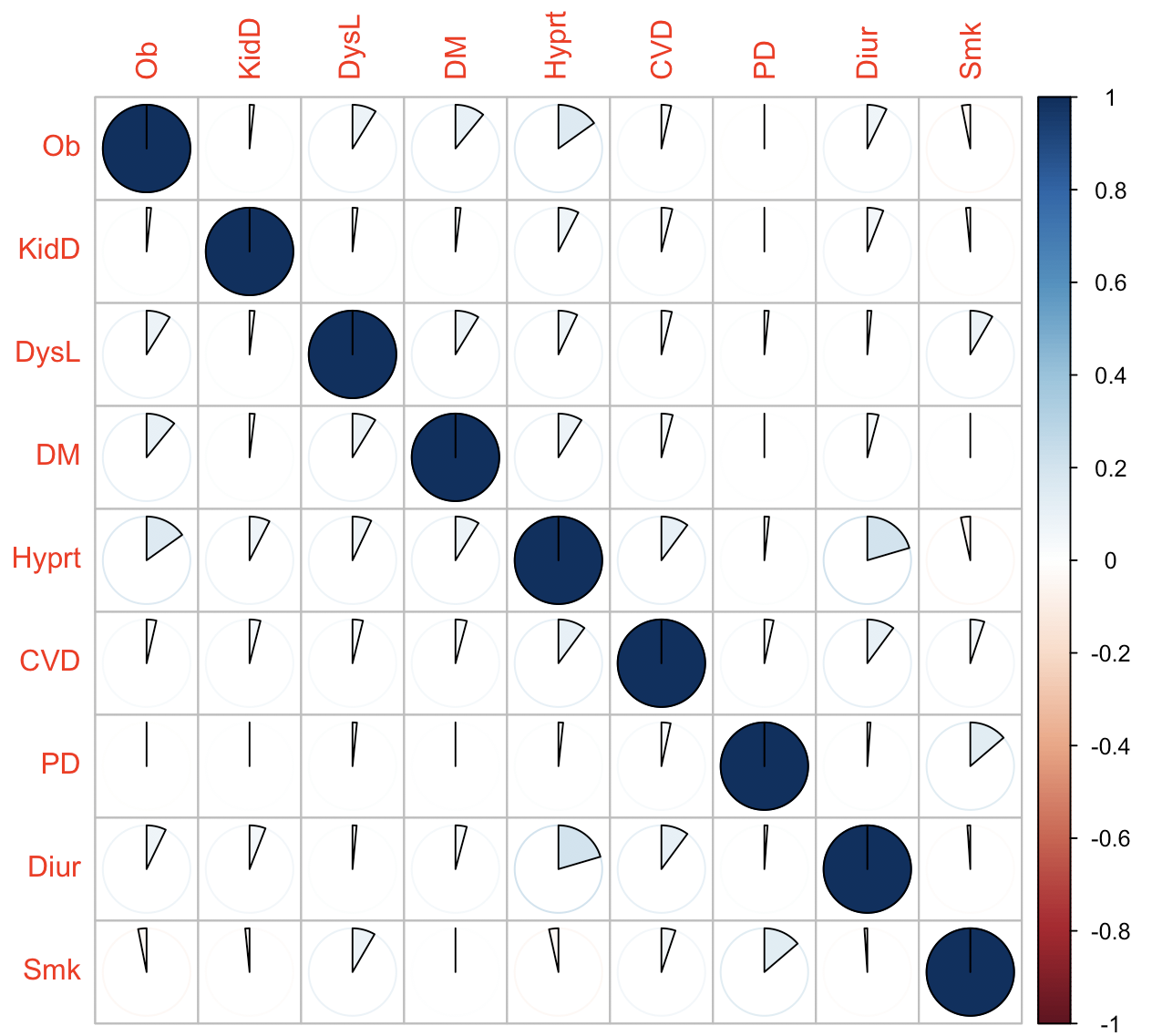


Figure S2 Pie chart indicating the degree of correlations (Pearson correlation) between variables included in the cluster analyses. The relative size of each sector in the pie charts represents the correlation coefficients. Ob: Obesity; KidD: Kidney dysfunction; DysL: Dys/hyperlipidaemia; DM: Diabetes mellitus; Hyprt: Hypertension; CVD: Cardiovascular disease; PD: Pulmonary dysfunction; Diur: Use of Diuretics; Smk: Smoking (current/previous).

**Table S1 ICD-codes used in National and regional Patient Register to identify subjects being diagnosed with different gout related comorbidities during follow-up**

| Diagnosis | ICD 10  1997- | ICD 9  1987-1996 | ICD 8  1968-1986 |
| --- | --- | --- | --- |
| Gout | M100-M104, M109 | 274 | 274 |
| Diabetes | E10-E14, O24 | 250, 648 | 250, 634.9, 661 |
| Obesity | E66 | 278 | 277 |
| Isolated hypercholesterolemia | E781 | 272B | 272, 279 |
| Isolated hypertriglyceridemia | E782 | 272C, 272H | 272.01, 272.8, 272.9, 273.8, 279.01 |
| Other hyperlipemia | E783-E785 | 272D, 272E | 279.2 |
| Hypertension | I10-I15 | 401-405 | 400-404, 412 |
| Ischemic heart disease | I20-I25 | 411-414 | 410-414 |
| Heart failure | I50 | 428-429 | 412-429, 458.9, 782.4, 997.1, 997.5, 997.6, 997.9 |
| Stroke/CVL/TIA | I60-I64, G45 | 430-436 | 430-436 |
| Renal disease/CKD | N00-N22 | 580W-592A | 580-592, 593.1, 593.2, 594, 792, 796 |
| Chronic obstructive pulmonary disease | J41-J44 | 491-496 | 491-493, 466, 491, 516-519, 783.3 |
| Alcoholism | Z721, F10 | V658, 291E, 292C, 305A | 291.9, 294.3, 303 |

ICD: International Classification of Diseases; CKD: Chronic kidney disease; CVL: Cerebrovascular lesion, TIA: Transient ischemic attack.

**Table S2 Descriptive information of participants of the MPP stratified by being included or excluded from the performed cluster analyses**

| Subset | Total number | Males | Females | Age (years) | BMI | Serum urate (µmol/L) |
| --- | --- | --- | --- | --- | --- | --- |
| All | 33335 | 22433 (67.3) | 10902 (32.7) | 45.68 ± 7.41 | 24.57 ± 3.62 | 300.77 ± 70.05 |
| Inlcuded in cluster analyses | 22057 | 14561 (66.0) | 7496 (34.0) | 46.81 ± 5.54 | 24.57 ± 3.53 | 298.73 ± 72.38 |
| Excluded from cluster analyses | 11278 | 7872 (69.8) | 3406 (30.2) | 43.35 ± 8.47 | 24.57 ± 3.68 | 304.77 ± 65.04 |

The characteristics are presented as mean ± standard deviation for continuous variables and number (percentages) for categorical variables.

**Table S3 Results of exploratory factor analysis using the nine selected comorbidities and lifestyle variables**

| **Comorbidity/Lifestyle variable** | **Factor numbers** | | | |
| --- | --- | --- | --- | --- |
|  | **F1** | | **F2** | **F3** |
| Use of diuretics | | **0.683** | -0.033 | -0.047 |
| Hypertension | | **0.617** | -0.092 | -0.324 |
| Cardiovascular disease | | **0.484** | 0.273 | -0.028 |
| Kidney dysfunction | | **0.422** | -0.012 | 0.087 |
| Smoking | | -0.073 | **0.755** | -0.059 |
| Pulmonary dysfunction | | 0.111 | **0.657** | 0.050 |
| Diabetes Mellitus | | 0.018 | -0.027 | **-0.621** |
| Dyslipidemia | | -0.080 | 0.251 | **-0.609** |
| Obesity | | 0.167 | -0.162 | **-0.608** |

The bold text represents the highest loading of the particular variable in corresponding factor. F1 to F3 represents factor numbers 1 to 3.

**Table S4 Mean SU values (µmol/L) by being diagnosed with gout or not in each separate cluster. Results are presented as mean difference in SU between the two sex groups**

| Cluster number | SU-mean (Males) | | | SU-mean (Females) | | |
| --- | --- | --- | --- | --- | --- | --- |
|  | **Gout**  ***(n=720)** | **No gout *(n=13819)** | **^Δ^Delta**  **(95%CI)** | **Gout**  ***(n=188)** | **No gout *(n=7304)** | **^Δ^Delta**  **(95%CI)** |
| C1 | 366.4 | 313.4 | 52.9  (46.9 to 58.9) | 270.2 | 238.8 | 31.3  (21.9 to 40.7) |
| C2 | 405.6 | 352.5 | 53.0  (27.4 to 78.6) | 349.25 | 275.5 | 73.7  (27.6 to 119.7) |
| C3 | 384.6 | 336.8 | 47.7  (11.0 to 84.4) | 295.5 | 265.3 | 30.1  (-13.2 to 73.4) |
| C4 | 392.2 | 342.3 | 49.91  (39.1 to 60.6) | 340.18 | 272.6 | 67.5  (46.1 to 89.0) |
| C5 | 421.2 | 340.8 | 80.3  (41.6 to 119.0) | 352.57 | 282.1 | 70.4  (37.5 to 103.2) |

*The difference in total number represents missing information for serum urate levels for 26 individuals (gout group = 2, non-gout group = 24). Δ represents the difference in mean serum urate in µmol/L, SU: serum urate, 95% CI: 95% confidence interval.

1. *Shared last authorship [↑](#footnote-ref-1)
